# Mechanistic Models of Signaling Pathways Reveal the Drug Action Mechanisms behind Gender-Specific Gene Expression for Cancer Treatments

**DOI:** 10.3390/cells9071579

**Published:** 2020-06-29

**Authors:** Cankut Çubuk, Fatma E. Can, María Peña-Chilet, Joaquín Dopazo

**Affiliations:** 1 Clinical Bioinformatics Area, Fundación Progreso y Salud (FPS), CDCA, Hospital Virgen del Rocio, 41013 Sevilla, Spain; c.cubuk@qmul.ac.uk (C.Ç.); fatmaezgican@gmail.com (F.E.C.); maria.pena.chilet.ext@juntadeandalucia.es (M.P.-C.); 2 Division of Genetics and Epidemiology, Institute of Cancer Research, London SW7 3RP, UK; 3 William Harvey Research Institute, Queen Mary University, London EC1M 6BQ, UK; 4 Department of Biostatistics, Faculty of Medicine, Izmir Katip Celebi University, 35620 Balatçık, Turkey; 5 Bioinformatics in Rare Diseases (BiER), Centro de Investigación Biomédica en Red de Enfermedades Raras (CIBERER), FPS, Hospital Virgen del Rocio, 41013 Sevilla, Spain; 6 Computational Systems Medicine, Institute of Biomedicine of Seville (IBIS), 41013 Sevilla, Spain; 7 FPS-ELIXIR-ES, Hospital Virgen del Rocío, 41013 Sevilla, Spain

**Keywords:** mechanistic models, gene expression, signaling pathways, signal transduction, cancer therapies, drug mechanism of action, gender bias

## Abstract

Despite the existence of differences in gene expression across numerous genes between males and females having been known for a long time, these have been mostly ignored in many studies, including drug development and its therapeutic use. In fact, the consequences of such differences over the disease mechanisms or the drug action mechanisms are completely unknown. Here we applied mechanistic mathematical models of signaling activity to reveal the ultimate functional consequences that gender-specific gene expression activities have over cell functionality and fate. Moreover, we also used the mechanistic modeling framework to simulate the drug interventions and unravel how drug action mechanisms are affected by gender-specific differential gene expression. Interestingly, some cancers have many biological processes significantly affected by these gender-specific differences (e.g., bladder or head and neck carcinomas), while others (e.g., glioblastoma or rectum cancer) are almost insensitive to them. We found that many of these gender-specific differences affect cancer-specific pathways or in physiological signaling pathways, also involved in cancer origin and development. Finally, mechanistic models have the potential to be used for finding alternative therapeutic interventions on the pathways targeted by the drug, which lead to similar results compensating the downstream consequences of gender-specific differences in gene expression.

## 1. Introduction

It has long been known that males and females present important differences that may influence the interpretation of traits, such as disease phenotypes [1,2] and their treatment [3]. In fact, since the introduction of microarrays, which allowed a systematic screening of the molecular differences between sexes, the existence of a large degree of sex-biased gene expression that could explain the molecular basis of such phenotypic differences became apparent [4]. However, in most studies, sex is ignored or is not properly taken into account despite a vast majority of common diseases displaying clear sex differences in symptoms or prevalence [5]. Reviews of studies based on animal models reveal an over-representation of experiments based exclusively on males [6]. Moreover, in many experiments including male and female animals, the results were not analyzed by sex [7,8]. In spite of this, it has been suggested that just adding sex as a variable could lead to conceptual and empirical errors in research unless differences between human men and women are properly modeled [9]. 

Thus, understanding the molecular basis of these differences is of utmost importance to identify the functional mechanisms behind them and being able to distinguish real sex-dependent cell activities from those ones due to confounding variables. Accordingly, in a recent study that revealed a strong gender-specific bias gene expression in osteoarthritis, conventional pathway enrichment analysis showed that female specific miRNAs were estrogen responsive and targeted genes in toll-like receptor signaling pathways, suggesting mechanistic links between inflammation and osteoarthritis [10]. In addition, recently, the discovery of differences in a brain signaling pathway involved in reward learning and motivation that make male mice more vulnerable to autism seems to provide a mechanistic explanation on why autism spectrum disorders are more common in males [11]. 

Therefore, a proper interpretation of the effect that differences in gene expression have over phenotypes, such as drug response or disease progression, involves understanding the mechanisms of the disease or the mode of action of drugs, which can be interpreted through mechanistic models of cell signaling [12] or cell metabolism [13]. Mechanistic models have helped to understand the disease mechanisms behind different cancers [14,15], including neuroblastoma [16,17], breast cancer [18], rare diseases [19], complex diseases [20], the mechanisms of action of drugs [21,22], and other biologically interesting scenarios such as the molecular mechanisms that explain how stress-induced activation of brown adipose tissue prevents obesity [23] or the molecular mechanisms of death and the post-mortem ischemia of a tissue [24]. Among the few available proposals of mechanistic modeling algorithms that model different aspects of signaling pathway activity, Hipathia has demonstrated having superior sensitivity and specificity [12]. 

Here, we propose the use of mechanistic models [13,14] of signaling activity related with cancer hallmarks [25], other cancer-related signaling pathways, and some extra relevant cellular functions to understand the functional consequences of the gender bias in gene expression. Such mechanistic models use gene expression data to produce an estimation of profiles of signaling or metabolic circuit activity within pathways [13,14]. An interesting property of mechanistic models is that they can be used not only to understand molecular mechanisms of disease or of drug action but also to predict the potential consequences of gene perturbations over the circuit activity in a given condition [26]. Actually, in a recent work, our group has successfully predicted therapeutic targets in cancer cell lines with a precision over 60% [15]. Therefore, we will use this mechanistic framework to understand what is the molecular basis of the different effects of cancer drugs by directly simulating their effect in the patients. This approach has recently been used by us to understand the generation of resistances in cancer at the single cell level in glioblastoma [27].

Therefore, circuit activity, which can easily be linked to specific cell functionalities, has been used here to discover the different molecular mechanisms triggered by the biased gene expression between human males and females in cancers and, what is even more interesting, in their differential response to treatments. 

## 2. Materials and Methods

### 2.1. Data Source, Selection Criteria, and Data Preprocessing

Gene expression data from patients belonging to The Cancer Anatomy Genome Project (TCGA) were downloaded from the International Cancer Genome Consortium (ICGC) data portal (https://dcc.icgc.org/).

To create datasets containing males and females with features as homogeneous and unbiased as possible, the Propensity Score Matching (PSM) technique [28] (MatchIt R package) was used. This methodology allows selecting samples that are different in gender but as similar as possible in the rest of the relevant features. To achieve so, first, a logistic regression model of gender (male/female) was created and regressed on the following covariates: age at initial pathologic diagnosis, histological type, pathologic stage, neoplasm histologic grade, race, tobacco smoking history, and tumor purity. All the covariates were taken from the ICGC data portal, except the tumor purity that was available at Synapse (https://www.synapse.org/#!Synapse:syn3242754). Next, the unbiased samples which have matching covariate weight profiles were selected.

The trimmed mean of M-values (TMM) method (18) was used for the normalization of gene expression data originally obtained as gene read counts of samples. Normalized samples were log-transformed and truncation by quantile 0.99 was applied. Batch effect was corrected with COMBAT [29]. Finally, the values were normalized between 0 and 1, as required by the signaling circuit activity mechanistic model [14].

### 2.2. Differential Gene Expression

To obtain differentially expressed genes (DEG) between conditions compared (normal versus cancer or male versus female), a negative binomial generalized log-linear model was used after gene expression normalization. The *p*-values were adjusted using the False Discovery Rate (FDR) method [30]. The edgeR package [31] was used for this purpose.

### 2.3. Rationale of the Signaling Circuit Activity Mechanistic Model

Circuit activities are modelled as described in [14]. Pathways in the Kyoto Encyclopedia of genes and Genomes (KEGG) repository [32] are used to define circuits that connect any possible receptor protein to specific effector proteins that are ultimately responsible for triggering cell activities. A total of 98 KEGG pathways involving a total of 3057 genes that form part of 4726 protein nodes were used to define a total of 1287 signaling circuits. Normalized gene expression values are used as proxies of protein activity [33,34,35]. The intensity value of signal transduced to the effector is estimated by starting with an initial signal with an arbitrary value of 1 in the receptor, which is propagated along the nodes of the signaling circuits according to the following recursive equation:

(1) $$S_{n}=\upsilon_{n}\cdot \left(1-\underset{s_{a}\in A}{\prod }\left(1-s_{a}\right)\right)\cdot \underset{s_{i}\in I}{\prod }\left(1-s_{i}\right)$$

where *S_n_* is the signal intensity for the current node *n*, *v_n_* is its normalized gene expression value, *A* is the set of activation signals (*s_a_*), arriving to the current node from activation edges, and *I* is the set of inhibitory signals (*s_i_*) arriving to the node from inhibition edges [14]. 

Here the Hipathia R/Bioconductor package (https://doi.org/doi:10.18129/B9.bioc.hipathia), which implements the Hipathia model, is used. Additionally, a web server implementation is also freely available at: http://hipathia.babelomics.org/.

### 2.4. Cell Functional Output Triggered by the Signaling Circuit 

The effector nodes at the end of the circuits trigger cell functionalities. The functionality of the circuit has been annotated as the function that the effector performs. Such functionalities have been taken from the Uniprot [36] annotations. In the case of ambiguity (e.g., the general term of apoptosis can refer to its activation or repression), the Uniprot annotations were refined by manual curation using more detailed Gene Ontology [37] annotations or Gene Cards [38] information on gene functionality.

### 2.5. Association of Signaling Circuits Activities to Cancer Hallmarks

As explained below, each effector is known to be associated with one or several cell functions. Since these effector genes have been related specifically with one or several cancer hallmarks [25] in the scientific literature, the CHAT tool [39], a text mining based application to organize and evaluate scientific literature on cancer, has been used to link gene names with cancer hallmarks. 

### 2.6. Estimation of the Differential Signaling Activity

The Hipathia R/Bioconductor package was used to test for differential signaling activity between male and female samples. Gene expression profiles are normalized as described above in Section 2.1 and uploaded in the Hipathia package. Then, these are transformed into the corresponding signaling circuit activity profiles, as explained above in Section 2.2. Finally, Hipathia applies a Wilcoxon test to check for significant differences in the activity of the circuits. The *p*-values are corrected for multiple testing using FDR [30]. 

### 2.7. Drug Effect Simulation

The effect that a drug with known targets has over the different signaling circuits is simulated using the PathAct [26] strategy. Briefly, the original gene expression profiles of the patients are taken as reference set and a simulated set of pseudo-treated patient gene expression profiles is generated by substituting the gene expression value(s) of the gene(s) targeted by the drug by a very low value (0.001) that simulates the inhibition of the drug. That is, the gene, even if it is expressed, is substituted by an “almost no expressed” gene (equivalent to an inhibited gene product. The reason for simulating the inhibition with an arbitrarily low value and not with a 0 is because in this way the simulation is more realistic (probably it is never an absolute inhibition) and, on the other hand, it preserves some basal low activity value in the circuit contributed by the rest of the genes, that it is useful for testing purposes (see [26] for details). Then, the HiPahtia R/Bioconductor package is used to generate the corresponding signaling profiles for the reference patient set and the pseudo-treated patient set, that are further compared and tested for differences with a Wilcoxon test. The *p*-values are corrected for multiple testing using FDR [30]. Table S1 contains the drugs that are used for each cancer in the simulation [40].

### 2.8. Differential Drug Effect between Male and Female Patients

In this case, for each cancer type and each drug, we will have two paired datasets of male and female patients untreated and with the simulation of the treatment. For each paired dataset, we can test whether the effect of the drug significantly affects any signaling circuit or not. However, we are looking for differences at circuit level when we compare the male versus the female datasets. Then, each paired comparison untreated versus simulated drug treatment produces a distribution of fold changes for each circuit in each patient. To check for gender-specific differences in drug treatments, we simply compare the mean fold change values obtained for male and female patients. 

## 3. Results

### 3.1. Data Processing

Gene expression matrices were downloaded from the ICGC data portal (https://dcc.icgc.org/) and processed as described in Methods. After the application of the PSM method to these data, a total of 3327 tumor samples corresponding to 13 different cancer types, containing samples of both genders with males and females with similar covariates, were used in the study (see Table 1). 

Profiles of normalized values of gene expression were then transformed into the corresponding profiles of signaling circuit activities upon the application of the Hipathia method [14] that can be used to detect Gender-Specific Differential Signaling Activity (GS-DSA) by testing significant differential signaling activity between males and females in each cancer type. DEG between cancer and normal samples were also estimated for the cancers as described in Methods. 

### 3.2. Gender-Specific Functional Differences in Cancer

**Figure 1:**
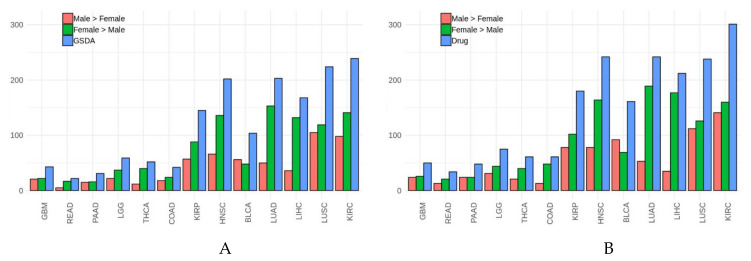
Number of signaling circuits with significant gender-specific differential signaling activity (GS-DSA) in the different cancer types studied. (**A**) the number of circuits with significant GS-DSA in each cancer, decomposed into those in which the activity of the signaling circuit is higher in males than in females and vice versa. (**B**) after simulation of the drug treatment the number of circuits showing significant GS-DSA increases.

While all cancer types contain signaling circuits with gender-specific differential behavior, the distribution in the number of these circuits is remarkably asymmetric (Figure 1). Specifically, READ, THCA, COAD, and GBM with 22, 52, 42, and 43 circuits, respectively, are cancers with a relatively small number of circuits with differential gender-specific activity, whereas, on the other extreme of the range, cancers like LUSC, KIRC, or HNSC with 224, 239, and 202 circuits, respectively (Table 2). Although for most cancers the number of circuits displaying a significant GS-DSA is similar among males and females, in three cancers HNSC, LUAD, and LIHC, and to a lesser extent also in THCA and KIRP, the number of circuits displaying significant signal activity differences is much higher in females than in males when the effect of the drug is simulated. 

**Figure 2:**
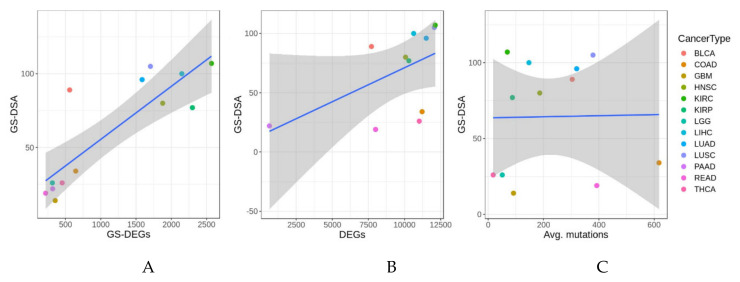
Relationships between. (**A**) gender-specific differential expressed genes (GS-DEG) and gender-specific differential signaling activity (GS-DSA); (**B**) differentially expressed genes between cancer and normal (DEG) and GS-DSA, and (**C**) average mutations (mutation burden) and GS-DSA.

While the number of signaling circuits showing differential gender-specific activity is proportional to the number of genes showing gender-specific differential expression (Figure 2A), this gender-specific differential signaling activity seems to be only slightly related to the level of differential expression between cancer and the normal tissue (Figure 2B) but completely unrelated to other relevant cancer parameters such as the mutational burden (Figure 2C). 

### 3.3. Potential Differences in Drug Effects Due to Gender-Specific Functional Differences

**Figure 3:**
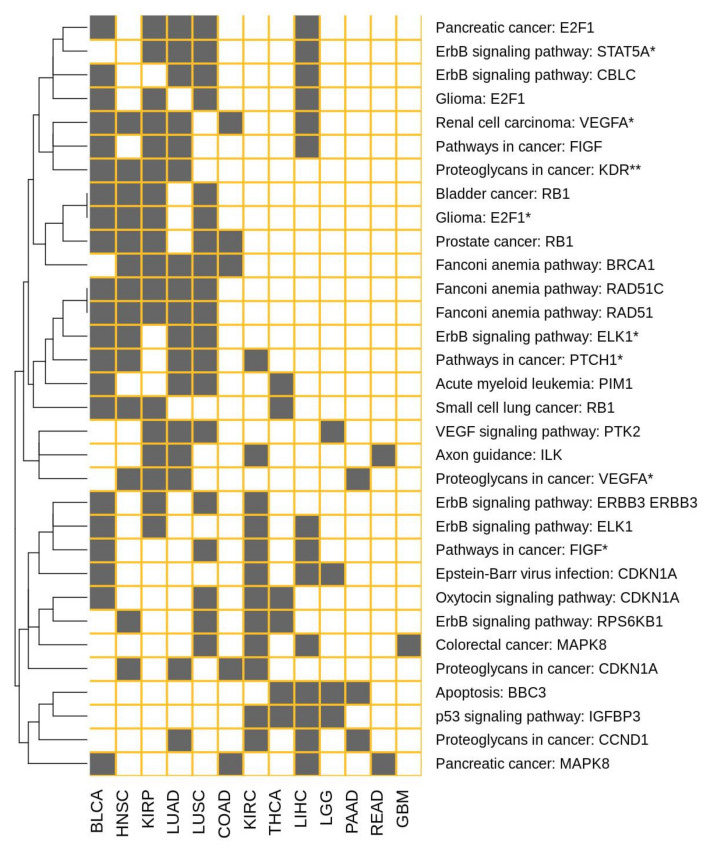
Distribution of the most pervasive GS-DSA circuits across cancer types. * and ** are used for disambiguation, it refers to effector genes occurring more than once in the same KEGG pathway.

**Figure 4:**
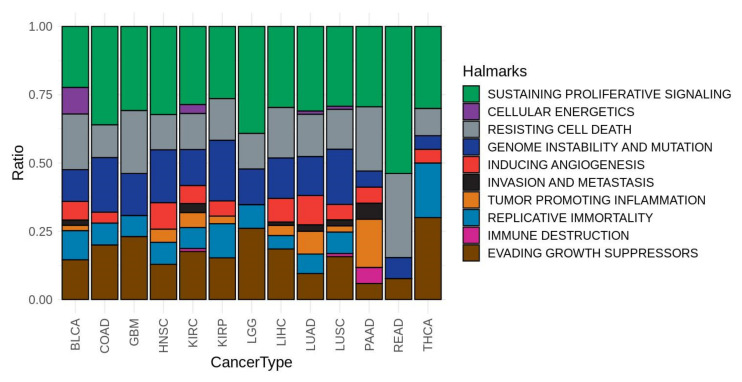
Cancer hallmarks affected by GS-DSA circuits across cancer types.

**Figure 5:**
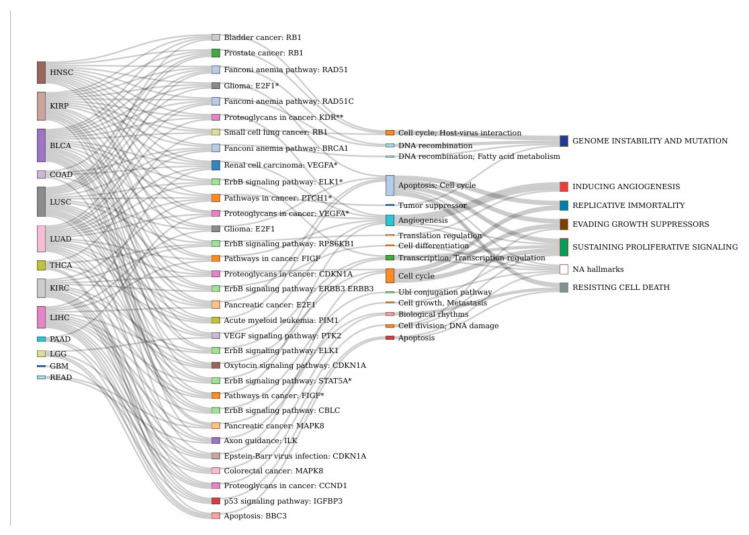
Relationships among cancers, signaling circuits, functions, and cancer hallmarks. * is used for disambiguation, it refers to effector genes occurring more than once in the same KEGG pathway.

In order to understand the molecular mechanisms behind gender-specific differential effect of drugs, their effect was simulated individually in each patient as described in Methods. Each cancer type studied (Table 1) was treated in the simulation with the specific drug(s) indicated (see Table S1). The simulation produced a new set of profiles of signaling activity corresponding to the simulated treatment for the patients. For both male and female patients, the simulated treatment sets were compared to the corresponding reference patient sets. Table S2 contains the circuits affected by the action of the different indicated drugs used in each cancer, both in male and female patients. Then, we were interested in circuits showing a significantly different effect of the drug between both sexes. The signaling circuits showing gender-specific differential behavior most pervasively across cancers occur only in a maximum of six cancer types simultaneously, which suggests a high heterogeneity in signaling programs across cancers. The cell functionalities triggered by the most pervasive gender-specific signaling circuits (presenting GS-DSA in at least four cancer types) are summarized in Table 3. Most of the affected circuits belong to cancer-specific pathways, such as renal cell carcinoma, pancreatic cancer, prostate cancer, glioma, etc. There are also some physiological pathways such as ErbB (KEGG: hsa04012), p53 (KEGG: hsa04115), Apoptosis (KEGG: hsa04210), or VEGF (KEGG: hsa04370) signaling pathways. The functionalities affected can easily be mapped to cancer hallmarks [25], such as angiogenesis, DNA recombination, Cell cycle, apoptosis, etc. Figure 3 depicts the distribution of the most pervasive GS-DSA circuits across cancer types. Figure 4 shows the cancer hallmarks most affected by the gender differences in gene expression and their consequences on signaling and ultimately in cell functionality. Table S2 contains a comprehensive list of all the circuits, with details on the cancer hallmarks affected. The distribution of circuits showing GS-DSA is uneven across cancer types, with cancers with many circuits affected, such as BCLA, HNSC of KIRP, and others with only a few circuits affected by the gender activity bias, such as GMB or READ. Finally, Figure 5 depicts a comprehensive map of relationships among cancers, signaling circuits, functions, and cancer hallmarks.

### 3.4. Validation

So far, the results depict the effects caused in signaling activity by the observed gender-biased differences in gene expression. However, the phenotypic consequences of these changes can be diverse in relevance and nature. In order to detect changes associated with drug effect, an exhaustive search in the literature has been done and for the following drugs a different activity in males and females was experimentally demonstrated: bevacizumab [41], cabozantinib [42], gefitinib [43], lapatinib [44], nilotinib [45], ruxolitinib [46], sorafenib [47], sunitinib [48], and trametinib [49]. In addition, for vemurafenib [50] and sonidegib [51], a low gender effect was also demonstrated, although not enough for different dose indications (see Table S1). Table 4 lists the circuits that display GS-DSA when the effect of the drug has been simulated. The simulation has been made with drugs known to have a different effect for males and females, and it is important to note that Table 4 only reports those circuits that were only differentially regulated in a drug with different activity between males and females and never in drugs which do not show this differential activity (see the whole list of drugs tested in Table S3). Figure S1 presents a comprehensive picture of the number of circuits affected and those that are relevant in the context of drug action. It is interesting to see how some drugs have an extensive gender-specific effect across many pathways, and, within them, across many signaling circuits, like ruxolitinib, while others seem to be circuit-specific, like bevacizumab or sorafenib. It is interesting to note that cancer-related pathways seem to be more pervasively affected by gender-specific differential activity in the drug simulation than physiologic pathways. 

## 4. Discussion

The differences associated with gender have been previously assessed in pan-cancer studies, most of them using TCGA cancer datasets, resulting in divergent patterns for sex bias in gene expression or immune features across multiple cancer types have been revealed [40,52]. Nevertheless, the functional consequences at the level of cell behavior or fate of gender bias in gene expression have remained mainly unknown. To our knowledge, this is the first time that such gender specific differences in gene expression are evaluated in the context of perturbation response, taking into consideration cell mechanisms as a whole, an approach that has successfully been used to explain different cancer molecular mechanisms [14,15,17,20,53].

Differences in cancer epidemiology, susceptibility, and prognostics have been widely described, but exactly why this occurs at a molecular level has been poorly understood. Many cancers show dissimilarities in incidence and mortality rates associated to sex-specific disparities; some can be the result of different hormone levels, especially estrogen, or sexual chromosome dose [54,55]. However, other differences, such as chemotherapy [56] or targeted therapy response [57,58], are the result of more complex cell processes that need to be evaluated in its cell mechanism context in order to be able to detect patterns. 

When evaluating individual circuits and pathways, most of them are indeed related with several cancer processes, apoptosis, and proliferation. Signaling circuits of the Fanconi Anemia pathway, involved in DNA repair [59], and therefore the genome instability and mutation cancer hallmark, show the highest values of GS-DSA. Other relevant signaling circuits belong to the proteoglycans in cancer pathways. Proteoglycans abundance, their metabolism and their relationship with genomic instability is clearly gender-related [60,61]. The ErbB signaling pathway, whose regulation is highly associated with estrogen and androgen levels [62,63] and Oxytocin signaling pathway, associated with vasopressin, a known sex dependent pathway [64], also contain signaling circuits showing significant GS-DSA.

In order to assess the gender-specific differences in global mechanisms across cancers, we grouped the circuits by cancer hallmark (Figure 4). Most of the hallmarks showed a gender-specific perturbation response pattern, but above all we find sustaining proliferative signaling, resisting cell death and evading growth suppressors hallmarks, mainly composed of circuits belonging to cell proliferation and apoptosis pathways. Indeed, gender-related differences in cell proliferation and differentiation pathways have already been described for several tissues in humans [65,66,67,68].

Genome instability hallmark shows a considerable number of GS-DSA circuits across cancers as well, which is concordant with previous studies, showing gender-associated differences in expression of genes involved in the aforementioned DNA repair [69,70], a different pattern of copy-number aberrations [71] or oxidative stress [72]. Moreover, available data suggest that sex influences measures of age-associated genomic instability, which increases in both males and females with age. However, how sex affects genome instability is less clear, as tissue studied, genetic background, and the method selected can influence results immensely, as well as environmental factors that are difficult to address [73].

Another cell process showing GS-DSA is lipid metabolism. It is well known that fat pad shows a different pattern in females and males, and these differences are the result of differences in metabolism at a molecular level [74,75,76]. Since some cancers present a high involvement of lipid metabolism in tumor initiation and progression, considering the intrinsic gender differences seems logical [77,78,79].

Besides the general deregulation of hallmarks, some of them are of special relevance in certain cancers. Particularly important is the role of angiogenesis, tumor promoting inflammation, and metastasis, which shows a clear pattern of GS-DSA in PAAD, KIRC, and LUAD, where some gender-specific events have already been described, as mutations [80,81], and in general, risk [82,83]. KIRC, together with HSNC and LUSC, are the cancers with the highest number of gender-specific signaling circuits, all of them showing sex-dependent differences in prognosis, mortality, and treatment response, as well as in molecular characteristics associated with them [71,84,85,86]. It is interesting to note that some cancers, such as PAAD, LUAD, and LUSC, are highly influenced by environmental factors, and therefore the gender differences might not be of physiological origin but rather could be determined by gender-specific lifestyles.

In order to evaluate the implications of gender in cancer, there are many factors that need to be addressed beyond the scope of this work, such as the possible implication of sex-biased transcription factors, miRNAs expression [87], methylation pattern [88,89], or even innate immune response [90,91]. However, no one can argue that biological intrinsic differences between females and males exist, and these differences are influencing all kind of cell behavior, thus the identification of the processes underlying these differences will facilitate the exploration of sex-biased disease susceptibility and therapy. 

Many of the proliferation-related circuits targeted by drugs presenting sex-bias are indeed regulated by estrogen in a direct or indirect manner, such as Ras, cGMP-PKG, or cAMP signaling and all the circuits related to MAPK proteins, as osteoclast differentiation [92,93]. Therefore, these circuits can show a different response depending on estrogen and other hormones levels, which are highly variable between sexes. Moreover, as aforementioned, lipid metabolism and proteoglycans may be influenced by hormone levels and by sex. Interestingly, melanogenesis, the synthesis of melanin and responsible of pigmentation, has been demonstrated to be regulated by hormones in a sex-specific manner in model organisms [94], and some studies suggest that these gender-associated pigmentation differences also occur in humans [95,96,97]. Moreover, skin hyperpigmentation is indeed more frequent in women and may be linked to sexual hormones [98,99].

The results presented here highlight the fact that gender needs to be considered when choosing the appropriate treatment in cancer. The approach presented here, based on mechanistic modeling of cell signaling pathways, shows its potential in evaluating the gender-specific differences in certain mechanisms of action of several drugs, and, therefore, in predicting potential non-responders and resistances [58,100]. It has also been shown that mechanistic models can be an excellent tool for the simulation of the effect of drugs, as we have recently demonstrated in cancer [27]. In particular, Table 4 shows circuits that display GS-DSA after the simulation of drugs for which a gender-specific activity has been reported, but never display GS-DSA in simulations of drugs with similar activity in both genders. In this way, the modeling framework used here provides the mechanistic link between the effect of the drug at a molecular level and at a phenotypic level. 

Like in any other study based on gene expression, it must be considered that any port-transcriptional modification, which can be relevant in cancer, is not primarily captured in the data. However, if such modification has an effect on the behavior of the neoplastic cell, it will be better detected, even indirectly, by its impact in the global signaling pattern of the cell, rather than by a conventional gene-centric analysis. In any case, mechanistic modeling can also be applied to proteomic or phosphoproteomic data, which would better account for the effect of post-transcriptional modifications in protein activity. However, given the difficulty of obtaining direct measurements of protein levels, an extensively used proxy for protein presence is the observation of the corresponding mRNA within the context of the module [34,53,101,102].

## 5. Conclusions

The use of mechanistic models that quantify cell behavioral outcomes provides a unique opportunity to understand the molecular mechanisms of cancer development and progression [103], and ultimately paves the way to suggest highly specific, individualized therapeutic interventions [26,104]. Here we demonstrate how mechanistic models are suitable for uncovering the functional consequences that the gender-biased gene expression triggers downstream signaling circuits. Mechanistic models offer an opportunity to reconsider alternative targets on the pathways relevant for the therapeutic interventions that lead to similar results compensating the downstream consequences of the gender-specific differences in gene expression.

## Figures and Tables

**Table 1:** Cancer types used in this study.

Cancer Code	Cancer Type	Female	Male	Sample Size	Proportion (Male/Female)
BLCA	Bladder urothelial carcinoma	57	202	259	3.54
COAD	Colon adenocarcinoma	113	207	320	1.83
GBM	Brain Glioblastoma Multiforme	37	89	126	2.41
HNSC	Head and Neck squamous cell carcinoma	97	328	425	3.38
KIRC	Kidney renal clear cell carcinoma	124	314	438	2.53
KIRP	Kidney renal papillary cell carcinoma	38	108	146	2.84
LGG	Brain Lower Grade Glioma	104	205	309	1.97
LIHC	Liver hepatocellular carcinoma	44	118	162	2.68
LUAD	Lung adenocarcinoma	131	213	344	1.63
LUSC	Lung squamous cell carcinoma	81	299	380	3.69
PAAD	Pancreatic Cancer	30	77	107	2.57
READ	Rectum adenocarcinoma	41	77	118	1.88
THCA	Thyroid Carcinoma	66	127	193	1.92
Total		963	2364	3327	

**Table 2:** Gender-specific differential gene expression and signaling circuit activation across cancers.

Cancer Type	Cancer	Cancer (M > F)	Cancer (F > M)	Drug Simulation	Drug (M > F)	Drug (F > M)	Drug Diff. Cancer
GBM	43	21	22	50	24	26	14
READ	22	5	17	34	13	21	19
PAAD	31	15	16	48	24	24	22
LGG	59	22	37	75	31	44	26
THCA	52	12	40	61	21	40	26
COAD	42	18	24	61	13	48	34
KIRP	145	57	88	180	78	102	77
HNSC	202	66	136	242	78	164	80
BLCA	104	56	48	161	92	69	89
LUAD	203	50	153	242	53	189	96
LIHC	168	36	132	212	35	177	100
LUSC	224	105	119	238	112	126	105
KIRC	239	98	141	301	141	160	107

**Table 3:** Circuits showing gender-specific differential signaling circuit activation in four or more cancers simultaneously.

Effector Circuit	Uniprot Annotation of Effector Circuits	Cancers with GS-DSA
Renal cell carcinoma: VEGFA *	Angiogenesis	BLCA, COAD, HNSC, KIRP, LIHC, LUAD
Fanconi anemia pathway: RAD51	DNA recombination	BLCA, HNSC, KIRP, LUAD, LUSC
Fanconi anemia pathway: RAD51C	DNA recombination	BLCA, HNSC, KIRP, LUAD, LUSC
Fanconi anemia pathway: BRCA1	DNA recombination;	COAD, HNSC, KIRP, LUAD, LUSC
Pathways in cancer: PTCH1 *	Tumor suppressor	BLCA, HNSC, KIRC, LUAD, LUSC
Pancreatic cancer: E2F1	Apoptosis; Cell cycle	BLCA, KIRP, LIHC, LUAD, LUSC
Prostate cancer: RB1	Cell cycle	BLCA, COAD, HNSC, KIRP, LUSC
ErbB signaling pathway: RPS6KB1	Translation regulation	HNSC, KIRC, LUSC, THCA
ErbB signaling pathway: ELK1	Transcription; Transcription regulation	BLCA, KIRC, KIRP, LIHC
ErbB signaling pathway: STAT5A *	Transcription; Transcription regulation	KIRP, LIHC, LUAD, LUSC
ErbB signaling pathway: ELK1 *	Transcription; Transcription regulation	BLCA, HNSC, LUAD, LUSC
ErbB signaling pathway: CBLC	Ubl conjugation pathway	BLCA, LIHC, LUAD, LUSC
ErbB signaling pathway: ERBB3 ERBB3	Cell differentiation	BLCA, KIRC, KIRP, LUSC
p53 signaling pathway: IGFBP3	Apoptosis	KIRC, LGG, LIHC, THCA
Apoptosis: BBC3	Apoptosis	LGG, LIHC, PAAD, THCA
Axon guidance: ILK	Cell growth, Metastasis	KIRC, KIRP, LUAD, READ
VEGF signaling pathway: PTK2	Angiogenesis	KIRP, LGG, LUAD, LUSC
Oxytocin signaling pathway: CDKN1A	Cell cycle	BLCA, KIRC, LUSC, THCA
Pathways in cancer: FIGF	Angiogenesis	BLCA, KIRP, LIHC, LUAD
Pathways in cancer: FIGF *	Angiogenesis	BLCA, KIRC, LIHC, LUSC
Proteoglycans in cancer: CCND1	Cell division; DNA damage	KIRC, LIHC, LUAD, PAAD
Proteoglycans in cancer: CDKN1A	Cell cycle	COAD, HNSC, KIRC, LUAD
Proteoglycans in cancer: VEGFA *	Angiogenesis	HNSC, KIRP, LUAD, PAAD
Proteoglycans in cancer: KDR **	Angiogenesis	BLCA, HNSC, KIRP, LUAD
Colorectal cancer: MAPK8	Biological rhythms	GBM, KIRC, LIHC, LUSC
Pancreatic cancer: MAPK8	Biological rhythms	BLCA, COAD, LIHC, READ
Glioma: E2F1	Apoptosis; Cell cycle	BLCA, KIRP, LIHC, LUSC
Glioma: E2F1 *	Apoptosis; Cell cycle	BLCA, HNSC, KIRP, LUSC
Bladder cancer: RB1	Cell cycle	BLCA, HNSC, KIRP, LUSC
Acute myeloid leukemia: PIM1	Apoptosis; Cell cycle	BLCA, LUAD, LUSC, THCA
Small cell lung cancer: RB1	Cell cycle	BLCA, HNSC, KIRP, THCA

* and ** are used for disambiguation, it refers to effector genes occurring more than once in the same KEGG pathway.

**Table 4:** Simulation of the effect that drugs, with described gender bias, have over signaling circuits (described as pathway and the final effector of the circuit). Circuits for which a GS-DSA is detected after the simulation of the drug are marked with “Y”.

Pathway	Effector	Bevacizumab	Cabozantinib	Gefitinib	Lapatinib	Nilotinib	Ruxolitinib	Sorafenib	Sunitinib	Trametinib	Vemurafenib	Sonidegib
Ras signaling pathway	BRAP	.	.	.	.	.	.	.	.	.	Y	.
cGMP-PKG signaling pathway	MAPK1	.	.	.	.	.	.	Y	.	.	.	.
cAMP signaling pathway	MYL9, PTCH1, HHIP, ACOX1, F2R AMH, ORAI1, BAD, NFKBIA NFKB1, RYR2, GRIN3A, GRIA1, CFTR, SLC9A1, ATP2B1, CACNA1C, PDE3A, ATP1B4 FXYD1, RHOA, C00165, C01245, PAK1, MLLT4, C00416, MAPK8, HCN4	.	.	.	.	.	Y	.	.	.	.	.
Chemokine signaling pathway	STAT1	.	.	.	.	.	Y	.	.	.	.	.
Wnt signaling pathway	JUN	.	.	Y	.	.	.	.	.	.	.	.
Hedgehog signaling pathway	PTCH1, SMO, PTCH1, GLI1, HHIP, CCND1, BCL2, PRKACA, GLI1 SUFU,	.	.	.	.	.	.	.	.	.	.	Y
Axon guidance	ILK	.	.	.	.	.	.	.	.	.	.	Y
VEGF signaling pathway:	NOS3	Y	.	.	.	.	.	.	.	.	.	.
Osteoclast differentiation:	MAPK1	.	.	.	.	.	.	.	Y	.	.	.
Osteoclast differentiation:	NFKB1	.	.	.	.	Y	.	.	.	.	.	.
Signaling pathways regulating pluripotency of stem cells	HNF1A	.	.	Y	.	.	.	.	.	.	.	.
Jak-STAT signaling pathway	BCL2, BCL2L1, MYC, AOX1, GFAP, MCL1, PIM1, CCND1	.	.	.	.	.	Y	.	.	.	.	.
Natural killer cell mediated cytotoxicity	TNF	.	.	.	.	.	.	.	.	Y	.	.
TNF signaling pathway	CASP7, JUN, CEBPB	.	.	.	.	.	.	.	.	Y	.	.
Leukocyte transendothelial migration	MAPK14	.	.	.	.	Y	.	.	.	.	.	.
Inflammatory mediator regulation of TRP channels:	TRPM8, TRPV4	.	.	.	.	.	Y	.	.	.	.	.
Ovarian steroidogenesis	STAR, HSD3B1, PLA2G4B, ACOT2, CYP19A1, HSD17B2, CYP19A1	.	.	.	.	.	Y	.	.	.	.	.
Melanogenesis	MITF	.	.	.	.	.	Y	.	.	.	.	.
Thyroid hormone synthesis	TG	.	.	.	.	.	Y	.	.	.	.	.
Thyroid hormone signaling pathway	STAT1, ESR1, THRB	.	.	.	.	.	.	.	.	Y	.	.
Adipocytokine signaling pathway	AGRP, NPY, POMC, PPARGC1A, PTPN11	.	.	.	.	.	Y	.	.	.	.	.
Regulation of lipolysis in adipocytes	PLIN1, LIPE	.	.	.	.	.	Y	.	.	.	.	.
Aldosterone synthesis and secretion	CYP11B2	.	.	.	.	.	Y	.	.	.	.	.
AGE-RAGE signaling pathway in diabetic complications	FOXO1, CCND1, NFATC1	.	.	.	.	Y	.	.	.	.	.	.
Pathways in cancer	CCND1	.	.	.	.	.	.	.	.	.	Y	.
Pathways in cancer	FIGF	Y	.	.	.	.	.	.	.	.	.	.
Pathways in cancer	CCNA1, CSF3R, CSF2RA, CSF1R	.	.	.	.	.	.	.	.	.	.	.
Pathways in cancer	CSF1R	.	.	.	.	.	.	.	Y	.	.	.
Pathways in cancer	BMP2, GLI1, HHIP, PTCH1	.	.	.	.	.	.	.	.	.	.	Y
Proteoglycans in cancer	HSPB2	.	.	.	.	Y	.	.	.	.	.	.
Proteoglycans in cancer:	AKT3	.	Y	Y	.	.	.	.	.	.	.	.
Proteoglycans in cancer:	PRKCA	.	.	.	.	.	.	Y		.	.	.
Colorectal cancer:	MAPK8	.	.	.	.	.	.	.	.	Y	.	.
Renal cell carcinoma	VEGFA	Y	.	.	.	.	.	.	.	.	.	.
Renal cell carcinoma	RAP1A	.	Y		.	.	.	.	.	.	.	.
Renal cell carcinoma	AKT3	.	Y	Y	.	.	.	.	.	.	.	.
Pancreatic cancer	RAC1	.	.	.	.	.	.	.	.	Y	.	.
Pancreatic cancer	C00416	.	.	.	.	.	.	.	.	Y	.	.
Basal cell carcinoma	PTCH1	.	.	.	.	.		.	.	.	.	Y
Acute myeloid leukemia	CCNA1, SPI1	.	.	.	.	.	.	.	.	.	.	.
Non-small cell lung cancer	FOXO3	.	.	.	Y	.	.	.	.	.	.	.

## Supplementary Materials

The following are available online at https://www.mdpi.com/2073-4409/9/7/1579/s1, Figure S1: Heatmap with the simulation of the effect of the drug on the circuits, Table S1: Drugs that are used for each cancer in the simulation; Table S2: Circuits affected by the action of the different indicated drugs used in each cancer. Table S3: Results of the simulations of drug effects over any individual circuit for the drugs with targets within the corresponding circuit.

## Abbreviations (Cancer Abbreviations are Listed in Table 1):

**Abbreviation**	**Meaning**
CHAT	Cancer Hallmarks Analytics Tool
DEG	Differentially Expressed Genes
FDR	False Discovery Rate
GS-DEG	Gender-Specific Differential Expressed Genes
GS-DSA	Gender-Specific Differential Signaling Activity
ICGC	International Cancer Genome Consortium
KEGG	Kyoto Encyclopedia of Genes and Genomes
PSM	Propensity Score Matching
TMM	Trimmed Mean of M-values

## References

[1] Flanagan K.L. (2014). Sexual dimorphism in biomedical research: A call to analyse by sex. Trans. R. Soc. Trop. Med. Hyg..

[2] Woodruff T. (2014). Sex, equity, and science. Proc. Natl. Acad. Sci. USA.

[3] Klein S.L., Schiebinger L., Stefanick M.L., Cahill L., Danska J., De Vries G.J., Kibbe M.R., McCarthy M.M., Mogil J.S., Woodruff T. (2015). Opinion: Sex inclusion in basic research drives discovery. Proc. Natl. Acad. Sci. USA.

[4] Rinn J.L., Snyder M. (2005). Sexual dimorphism in mammalian gene expression. Trends Genet..

[5] Ober C., Loisel D.A., Gilad Y. (2008). Sex-specific genetic architecture of human disease. Nat. Rev. Genet..

[6] Karp N.A., Mason J., Beaudet A.L., Benjamini Y., Bower L., Braun R.E., Brown S.D., Chesler E.J., Dickinson M.E., Flenniken A.M. (2017). Prevalence of sexual dimorphism in mammalian phenotypic traits. Nat. Commun..

[7] Yoon D.Y., Mansukhani N.A., Stubbs V.C., Helenowski I.B., Woodruff T., Kibbe M.R. (2014). Sex bias exists in basic science and translational surgical research. Surgery.

[8] Beery A.K., Zucker I. (2010). Sex bias in neuroscience and biomedical research. Neurosci. Biobehav. Rev..

[9] Richardson S.S., Reiches M., Shattuck-Heidorn H., LaBonte M.L., Consoli T. (2015). Opinion: Focus on preclinical sex differences will not address women’s and men’s health disparities. Proc. Natl. Acad. Sci. USA.

[10] Kolhe R., Hunter M., Liu S., Jadeja R.N., Pundkar C., Mondal A.K., Mendhe B., Drewry M., Rojiani M.V., Liu Y. (2017). Gender-specific differential expression of exosomal miRNA in synovial fluid of patients with osteoarthritis. Sci. Rep..

[11] Grissom N.M., E McKee S., Schoch H., Bowman N., Havekes R., O’Brien W.T., Mahrt E., Siegel S., Commons K.G., Portfors C. (2017). Male-specific deficits in natural reward learning in a mouse model of neurodevelopmental disorders. Mol. Psychiatry.

[12] Amadoz A., Hidalgo M., Cubuk C., Caballero J.C., Dopazo J. (2019). A comparison of mechanistic signaling pathway activity analysis methods. Briefings Bioinform..

[13] Cubuk C., Hidalgo M.R., Amadoz A., Rian K., Salavert F., Pujana M.A., Mateo F., Herranz C., Caballero J.C., Dopazo J. (2018). Differential metabolic activity and discovery of therapeutic targets using summarized metabolic pathway models. bioRxiv.

[14] Hidalgo M.R., Cubuk C., Amadoz A., Salavert F., Caballero J.C., Dopazo J. (2016). High throughput estimation of functional cell activities reveals disease mechanisms and predicts relevant clinical outcomes. Oncotarget.

[15] Cubuk C., Hidalgo M., Amadoz A., Pujana M.A., Mateo F., Herranz C., Caballero J.C., Dopazo J. (2018). Gene Expression Integration into Pathway Modules Reveals a Pan-Cancer Metabolic Landscape. Cancer Res..

[16] Fey D., Halász M., Dreidax D., Kennedy S.P., Hastings J.F., Rauch N., Munoz A.G., Pilkington R., Fischer M., Westermann F. (2015). Signaling pathway models as biomarkers: Patient-specific simulations of JNK activity predict the survival of neuroblastoma patients. Sci. Signal..

[17] Hidalgo M.R., Amadoz A., Cubuk C., Carbonell-Caballero J., Dopazo J. (2018). Models of cell signaling uncover molecular mechanisms of high-risk neuroblastoma and predict disease outcome. Biol. Direct.

[18] Jiao Y., Hidalgo M., Cubuk C., Amadoz A., Caballero J.C., Vert J.-P., Dopazo J. (2017). Signaling Pathway Activities Improve Prognosis for Breast Cancer. bioRxiv.

[19] Chacón-Solano E., León C., Díaz F., García-García F., García M., Escámez M., Guerrero-Aspizua S., Conti C., Mencía Á., Martínez-Santamaría L. (2019). Fibroblast activation and abnormal extracellular matrix remodelling as common hallmarks in three cancer-prone genodermatoses. Br. J. Dermatol..

[20] Peña-Chilet M., Esteban-Medina M., Falco M.M., Rian K., Hidalgo M.R., Loucera C., Dopazo J. (2019). Using mechanistic models for the clinical interpretation of complex genomic variation. Sci. Rep..

[21] Amadoz A., Sebastian-Leon P., Vidal E., Salavert F., Dopazo J. (2015). Using activation status of signaling pathways as mechanism-based biomarkers to predict drug sensitivity. Sci. Rep..

[22] Esteban-Medina M., Peña-Chilet M., Loucera C., Dopazo J. (2019). Exploring the druggable space around the Fanconi anemia pathway using machine learning and mechanistic models. BMC Bioinform..

[23] Razzoli M., Frontini A., Gurney A., Mondini E., Cubuk C., Katz L.S., Cero C., Bolan P.J., Dopazo J., Vidal-Puig A. (2015). Stress-induced activation of brown adipose tissue prevents obesity in conditions of low adaptive thermogenesis. Mol. Metab..

[24] Ferreira P., Muñoz-Aguirre M., Reverter F., Godinho C.P.S., Sousa A., Amadoz A., Sodaei R., Hidalgo M.R., Pervouchine D., Carbonell-Caballero J. (2018). The effects of death and post-mortem cold ischemia on human tissue transcriptomes. Nat. Commun..

[25] Hanahan D., A Weinberg R. (2011). Hallmarks of Cancer: The Next Generation. Cell.

[26] Salavert F., Hidago M.R., Amadoz A., Cubuk C., Medina I., Crespo D., Carbonell-Caballero J., Dopazo J. (2016). Actionable pathways: Interactive discovery of therapeutic targets using signaling pathway models. Nucleic Acids Res..

[27] Falco M.M., Pena-Chilet M., Loucera C., Hidalgo M., Dopazo J. (2019). Mechanistic models of signaling pathways deconvolute the functional landscape of glioblastoma at single cell resolution. bioRxiv.

[28] Ho D.E., Imai K., King G., Stuart E.A. (2007). Matching as Nonparametric Preprocessing for Reducing Model Dependence in Parametric Causal Inference. Polit. Anal..

[29] Johnson W.E., Li C., Rabinovic A. (2006). Adjusting batch effects in microarray expression data using empirical Bayes methods. Biostatistics.

[30] Benjamini Y., Yekutieli D. (2001). The control of false discovery rate in multiple testing under dependency. Ann. Stat..

[31] Robinson M.D., McCarthy D.J., Smyth G.K. (2009). edgeR: A Bioconductor package for differential expression analysis of digital gene expression data. Bioinformatics.

[32] Kanehisa M., Goto S., Sato Y., Kawashima M., Furumichi M., Tanabe M. (2013). Data, information, knowledge and principle: Back to metabolism in KEGG. Nucleic Acids Res..

[33] Sebastian-Leon P., Vidal E., Minguez P., Conesa A., Tarazona S., Amadoz A., Armero C., Salavert F., Vidal-Puig A., Montaner D. (2014). Understanding disease mechanisms with models of signaling pathway activities. BMC Syst. Biol..

[34] Efroni S., Schaefer C.F., Buetow K.H. (2007). Identification of Key Processes Underlying Cancer Phenotypes Using Biologic Pathway Analysis. PLoS ONE.

[35] Montaner D., Minguez P., Al-Shahrour F., Dopazo J. (2009). Gene set internal coherence in the context of functional profiling. BMC Genom..

[36] UniProt Consortium (2014). The UniProt Consortium UniProt: A hub for protein information. Nucleic Acids Res..

[37] Carbon S., Douglass E., Dunn N., Good B., Harris N.L., Lewis S.E., Mungall C.J., Basu S., Chisholm R.L., The Gene Ontology Consortium (2018). The Gene Ontology Resource: 20 years and still GOing strong. Nucleic Acids Res..

[38] Stelzer G., Rosen N., Plaschkes I., Zimmerman S., Twik M., Fishilevich S., Stein T.I., Nudel R., Lieder I., Mazor Y. (2016). The GeneCards Suite: From Gene Data Mining to Disease Genome Sequence Analyses. Curr. Protoc. Bioinform..

[39] Baker S., Ali I., Silins I., Pyysalo S., Guo Y., Högberg J., Stenius U., Korhonen A. (2017). Cancer Hallmarks Analytics Tool (CHAT): A text mining approach to organize and evaluate scientific literature on cancer. Bioinformatics.

[40] Yuan Y., Liu L., Chen H., Wang Y., Xu Y., Mao H., Li J., Mills G.B., Shu Y., Li L. (2016). Comprehensive Characterization of Molecular Differences in Cancer between Male and Female Patients. Cancer Cell.

[41] Assessment Report MVASI. https://www.ema.europa.eu/en/documents/assessment-report/mvasi-epar-public-assessment-report_en.pdf.

[42] CHPM Assessment Report cabometyx. https://www.ema.europa.eu/en/documents/assessment-report/cabometyx-epar-public-assessment-report_en.pdf.

[43] Assessment Report for iressa. https://www.ema.europa.eu/en/documents/assessment-report/iressa-epar-public-assessment-report_en.pdf.

[44] Assessment Report for tyverb. https://www.ema.europa.eu/en/documents/assessment-report/tyverb-epar-public-assessment-report_en.pdf.

[45] Flagg P.J. (1942). Scientific Discussions. Anesthesiology.

[46] CHMP Assessment Report ruxolitinib. https://www.ema.europa.eu/en/documents/assessment-report/jakavi-epar-public-assessment-report_en.pdf.

[47] CHMP Extension of Indication Variation Assessment Report. https://www.ema.europa.eu/en/documents/variation-report/nexavar-h-c-690-ii-35-epar-assessment-report-variation_en.pdf.

[48] Segarra I., Modamio P., Fernández C., Mariño E.L. (2017). Sex-Divergent Clinical Outcomes and Precision Medicine: An Important New Role for Institutional Review Boards and Research Ethics Committees. Front. Pharmacol..

[49] CHMP Assessment Report trametinib. https://www.ema.europa.eu/en/documents/assessment-report/mekinist-epar-public-assessment-report_en.pdf.

[50] Assessment Report Zelboraf. https://www.ema.europa.eu/en/documents/assessment-report/zelboraf-epar-public-assessment-report_en.pdf.

[51] Assessment Report Odomzo. https://www.ema.europa.eu/en/documents/assessment-report/odomzo-epar-public-assessment-report_en.pdf.

[52] Ye Y., Jing Y., Li L., Mills G.B., Diao L., Liu H., Han L. (2020). Sex-associated molecular differences for cancer immunotherapy. Nat. Commun..

[53] Cubuk C., Hidalgo M.R., Amadoz A., Rian K., Salavert F., Pujana M.A., Mateo F., Herranz C., Carbonell-Caballero J., Dopazo J. (2019). Differential metabolic activity and discovery of therapeutic targets using summarized metabolic pathway models. NPJ Syst. Biol. Appl..

[54] Kim H.-I., Lim H., Moon A. (2018). Sex Differences in Cancer: Epidemiology, Genetics and Therapy. Biomol. Ther..

[55] Dorak M.T., Karpuzoglu E. (2012). Gender Differences in Cancer Susceptibility: An Inadequately Addressed Issue. Front. Genet..

[56] Özdemir B.C., Csajka C., Dotto G.-P., Wagner A.D. (2018). Sex Differences in Efficacy and Toxicity of Systemic Treatments: An Undervalued Issue in the Era of Precision Oncology. J. Clin. Oncol..

[57] A Pinto J., Vallejos C.S., E Raez L., A Mas L., Ruiz R., Torres-Roman J.S., Morante Z., Araujo J.M., Gomez H.L., Aguilar A. (2018). Gender and outcomes in non-small cell lung cancer: An old prognostic variable comes back for targeted therapy and immunotherapy?. ESMO Open.

[58] Wang S., Cowley L.A., Liu X.-S. (2019). Sex Differences in Cancer Immunotherapy Efficacy, Biomarkers, and Therapeutic Strategy. Molecules.

[59] Niedernhofer L.J., Lalai A.S., Hoeijmakers J.H. (2005). Fanconi Anemia (Cross)linked to DNA Repair. Cell.

[60] Gupta V., Barzilla J.E., Mendez J.S., Stephens E.H., Lee E.L., Collard C.D., Laucirica R., Weigel P.H., Grande-Allen K.J. (2008). Abundance and location of proteoglycans and hyaluronan within normal and myxomatous mitral valves. Cardiovasc. Pathol..

[61] Oh J.-H., Kim Y.K., Jung J.-Y., Shin J.-E., Chung J.H. (2011). Changes in glycosaminoglycans and related proteoglycans in intrinsically aged human skin in vivo. Exp. Dermatol..

[62] Levin E.R. (2003). Bidirectional Signaling between the Estrogen Receptor and the Epidermal Growth Factor Receptor. Mol. Endocrinol..

[63] Bonaccorsi L. (2004). The androgen receptor associates with the epidermal growth factor receptor in androgen-sensitive prostate cancer cells. Steroids.

[64] Carter C.S. (2017). The Oxytocin–Vasopressin Pathway in the Context of Love and Fear. Front. Endocrinol..

[65] A Fitzpatrick L., Ruan M., Anderson J., Moraghan T., Miller V. (1999). Gender-related differences in vascular smooth muscle cell proliferation: Implications for prevention of arteriosclerosis. Lupus.

[66] Kerksick C.M., Taylor L., Harvey A., Willoughby D. (2008). Gender-Related Differences in Muscle Injury, Oxidative Stress, and Apoptosis. Med. Sci. Sports Exerc..

[67] Fossett E., Khan W.S., Longo U.G., Smitham P. (2012). Effect of age and gender on cell proliferation and cell surface characterization of synovial fat pad derived mesenchymal stem cells. J. Orthop. Res..

[68] Mallat Z., Fornes P., Costagliola R., Esposito B., Belmin J., LeComte D., Tedgui A. (2001). Age and gender effects on cardiomyocyte apoptosis in the normal human heart. J. Gerontol. Ser. A Biol. Sci. Med. Sci..

[69] Zhang J., Yan S., Liu X., Gan L., Wu Z., Gong Y., Huang M., Zhang X., Guo W. (2017). Gender-related prognostic value and genomic pattern of intra-tumor heterogeneity in colorectal cancer. Carcinogenesis.

[70] Köglsberger S., Cordero-Maldonado M.L., Antony P., Forster J.I., Garcia P., Buttini M., Crawford A., Glaab E. (2016). Gender-Specific Expression of Ubiquitin-Specific Peptidase 9 Modulates Tau Expression and Phosphorylation: Possible Implications for Tauopathies. Mol. Neurobiol..

[71] Li C.H., Haider S., Shiah Y.-J., Thai K., Boutros P.C. (2018). Sex Differences in Cancer Driver Genes and Biomarkers. Cancer Res..

[72] Ali I., Högberg J., Hsieh J.-H., Auerbach S., Korhonen A., Stenius U., Silins I. (2016). Gender differences in cancer susceptibility: Role of oxidative stress. Carcinogenesis.

[73] Fischer K.E., Riddle N.C. (2018). Sex Differences in Aging: Genomic Instability. J. Gerontol. Ser. A Biol. Sci. Med. Sci..

[74] Jensen M.D. (1995). Gender differences in regional fatty acid metabolism before and after meal ingestion. J. Clin. Investig..

[75] Childs C.E., Romeu-Nadal M., Burdge G.C., Calder P.C. (2008). Gender differences in the n-3 fatty acid content of tissues. Proc. Nutr. Soc..

[76] Mittendorfer B. (2005). Sexual Dimorphism in Human Lipid Metabolism. J. Nutr..

[77] Santos C.R., Schulze A. (2012). Lipid metabolism in cancer. FEBS J..

[78] Long J., Zhang C.-J., Zhu N., Du K., Yin Y.-F., Tan X., Liao D.-F., Qin L. (2018). Lipid metabolism and carcinogenesis, cancer development. Am. J. Cancer Res..

[79] Munir R., Lisec J., Swinnen J.V., Zaidi N. (2019). Lipid metabolism in cancer cells under metabolic stress. Br. J. Cancer.

[80] Ricketts C.J., Linehan W.M. (2015). Gender Specific Mutation Incidence and Survival Associations in Clear Cell Renal Cell Carcinoma (CCRCC). PLoS ONE.

[81] Tseng C.-H., Chiang C.-J., Tseng J.-S., Yang T.-Y., Hsu K.-H., Chen K.-C., Wang C.-L., Chen C.-Y., Yen S.-H., Tsai C.-M. (2017). EGFR mutation, smoking, and gender in advanced lung adenocarcinoma. Oncotarget.

[82] Andersson G., Wennersten C., Borgquist S., Jirström K. (2016). Pancreatic cancer risk in relation to sex, lifestyle factors, and pre-diagnostic anthropometry in the Malmö Diet and Cancer Study. Biol. Sex. Differ..

[83] Rawla P., Sunkara T., Gaduputi V. (2019). Epidemiology of Pancreatic Cancer: Global Trends, Etiology and Risk Factors. World J. Oncol..

[84] Haake S.M., Brannon A.R., Hacker K., Pruthi R., Wallen E., Nielsen M.E., Rathmell K. (2012). Use of meta-analysis of clear cell renal cell carcinoma gene expression to define a variant subgroup and identify gender influences on tumor biology. J. Clin. Oncol..

[85] Lee S.H., Oh S.-Y., I Do S., Lee H.J., Kang H.J., Rho Y.S., Bae W.J., Lim Y.C. (2014). SOX2 regulates self-renewal and tumorigenicity of stem-like cells of head and neck squamous cell carcinoma. Br. J. Cancer.

[86] Hu Z., Wu J., Lai S., Xu Y., Zhan J., Li R., Liu X., Wang N., Wei X., Jiang X. (2020). Clear cell renal cell carcinoma: The value of sex-specific abdominal visceral fat measured on CT for prediction of Fuhrman nuclear grade. Eur. Radiol..

[87] Cui C., Yang W., Shi J., Zhou Y., Yang J., Cui Q., Zhou Y. (2018). Identification and Analysis of Human Sex-biased MicroRNAs. Genom. Proteom. Bioinform..

[88] Liu J., Morgan M., Hutchison K., Calhoun V.D. (2010). A Study of the Influence of Sex on Genome Wide Methylation. PLoS ONE.

[89] Boks M.P.M., Derks E.M., Weisenberger D.J., Strengman E., Janson E., Sommer I.E.C., Kahn R.S., Ophoff R.A. (2009). The Relationship of DNA Methylation with Age, Gender and Genotype in Twins and Healthy Controls. PLoS ONE.

[90] O’Brown Z.K., Van Nostrand E., Higgins J.P., Kim S.K. (2015). The Inflammatory Transcription Factors NFκB, STAT1 and STAT3 Drive Age-Associated Transcriptional Changes in the Human Kidney. PLoS Genet..

[91] Fan H., Dong G., Zhao G., Liu Z., Yao G., Zhu Y., Hou Y. (2014). Gender Differences of B Cell Signature in Healthy Subjects Underlie Disparities in Incidence and Course of SLE Related to Estrogen. J. Immunol. Res..

[92] Atanaskova N., Keshamouni V., Krueger J.S., A Schwartz J., Miller F., Reddy K. (2002). MAP kinase/estrogen receptor cross-talk enhances estrogen-mediated signaling and tumor growth but does not confer tamoxifen resistance. Oncogene.

[93] Driggers P.H., Segars J. (2002). Estrogen action and cytoplasmic signaling pathways. Part II: The role of growth factors and phosphorylation in estrogen signaling. Trends Endocrinol. Metab..

[94] Guillot R., Muriach B., Rocha A., Rotllant J., Kelsh R.N., Cerdá-Reverter J.M. (2016). Thyroid Hormones Regulate Zebrafish Melanogenesis in a Gender-Specific Manner. PLoS ONE.

[95] Martinez-Cadenas C., Peña-Chilet M., Ibarrola-Villava M., Ribas G. (2013). Gender is a major factor explaining discrepancies in eye colour prediction based on HERC2/OCA2 genotype and the IrisPlex model. Forensic Sci. Int. Genet..

[96] Pietroni C., Andersen M.M., Johansen P., Andersen M.M., Harder S., Paulsen R.R., Børsting C., Morling N. (2014). The effect of gender on eye colour variation in European populations and an evaluation of the IrisPlex prediction model. Forensic Sci. Int. Genet..

[97] Hernando B., Ibarrola-Villava M., Fernández L.P., Pena-Chilet M., Llorca-Cardeñosa M., Oltra S.S., Alonso S., Boyano M.D., Cadenas C.M., Ribas G. (2016). Sex-specific genetic effects associated with pigmentation, sensitivity to sunlight, and melanoma in a population of Spanish origin. Biol. Sex. Differ..

[98] Thornton M.J. (2002). The biological actions of estrogens on skin. Exp. Dermatol..

[99] Lee A.-Y. (2015). Recent progress in melasma pathogenesis. Pigment. Cell Melanoma Res..

[100] Hohla F., Hopfinger G., Romeder F., Rinnerthaler G., Bezan A., Stättner S., Hauser-Kronberger C., Ulmer H., Greil R. (2013). Female gender may predict response to FOLFIRINOX in patients with unresectable pancreatic cancer: A single institution retrospective review. Int. J. Oncol..

[101] Sebastian-Leon P., Carbonell J., Salavert F., Sanchez R., Medina I., Dopazo J. (2013). Inferring the functional effect of gene expression changes in signaling pathways. Nucleic Acids Res..

[102] Mitsos A., Melas I., Siminelakis P., Chairakaki A.D., Saez-Rodriguez J., Alexopoulos L.G. (2009). Identifying Drug Effects via Pathway Alterations using an Integer Linear Programming Optimization Formulation on Phosphoproteomic Data. PLoS Comput. Biol..

[103] Fryburg D.A., Song D.H., Laifenfeld D., De Graaf D. (2014). Systems diagnostics: Anticipating the next generation of diagnostic tests based on mechanistic insight into disease. Drug Discov. Today.

[104] Dopazo J. (2014). Genomics and transcriptomics in drug discovery. Drug Discov. Today.

