# Supplementary figures and images for: Mechanistic Models of Signaling Pathways Reveal the Drug Action Mechanisms behind Gender-Specific Gene Expression for Cancer Treatments
Source: Cells. 2020 Jun 29;9(7):1579. doi: 10.3390/cells9071579 (PMC7408716; doi:10.3390/cells9071579)

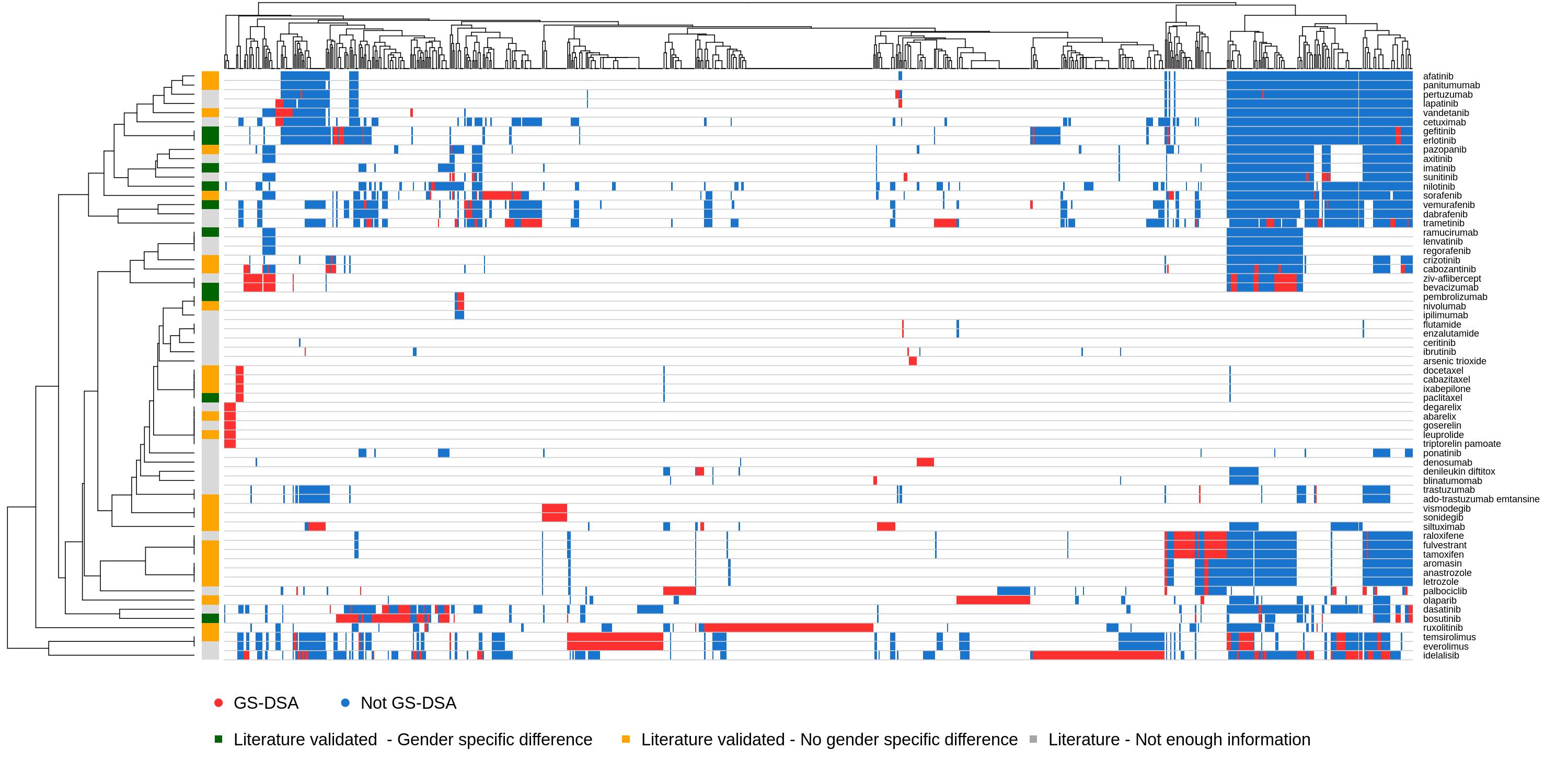

Supplement: Supplementary file 1 [file cells-09-01579-s001.zip › Figure S1.jpeg]
